# Magnification effects on perspective angle and optical slant angle across locations

**DOI:** 10.1177/20416695251351412

**Published:** 2025-07-03

**Authors:** Peeraya Sripian, Takashi Ijiri, Yasushi Yamaguchi

**Affiliations:** 1 College of Engineering, Shibaura Institute of Technology, Tokyo, Japan; 2 Graduate School of Arts and Sciences, The University of Tokyo, Tokyo, Japan

**Keywords:** perception, spatial vision, depth, binocular vision

## Abstract

This study investigates the phenomenon of magnification illusion, where the perception of perspective and optical slant angles changes when a scene is magnified. Our findings indicate that magnification influences these angles differently depending on location, which suggests that the illusion might be caused by changes in three-dimensional (3D) interpretation. Our findings reveal that the change of perspective angle interpretation primarily occurred when the stimuli were on the ground and sidewall but not those on the ceiling. Specifically, stimuli on the ceiling exhibit a significant underestimation of optical slant angles, while the perspective angle remains relatively stable. We developed a mathematical model based on the hypothesis of changes in 3D interpretation, which aligns well with our data. It was found that the interpretation of the perspective angle and the optical slant angle changes when a scene is magnified as indicated by the proposed relationship. This research provides the characteristics underlying spatial perception and its alteration under magnification and relative location, with potential applications in virtual reality and augmented reality system designs.

## How to cite this article

Sripian, P., Ijiri, T., & Yamaguchi, Y. (2025). Magnification effects on perspective angle and optical slant angle across locations. i-Perception, 16(4), 1–26. https://doi.org/10.1177/20416695251351412

## Introduction

**Figure 1.**
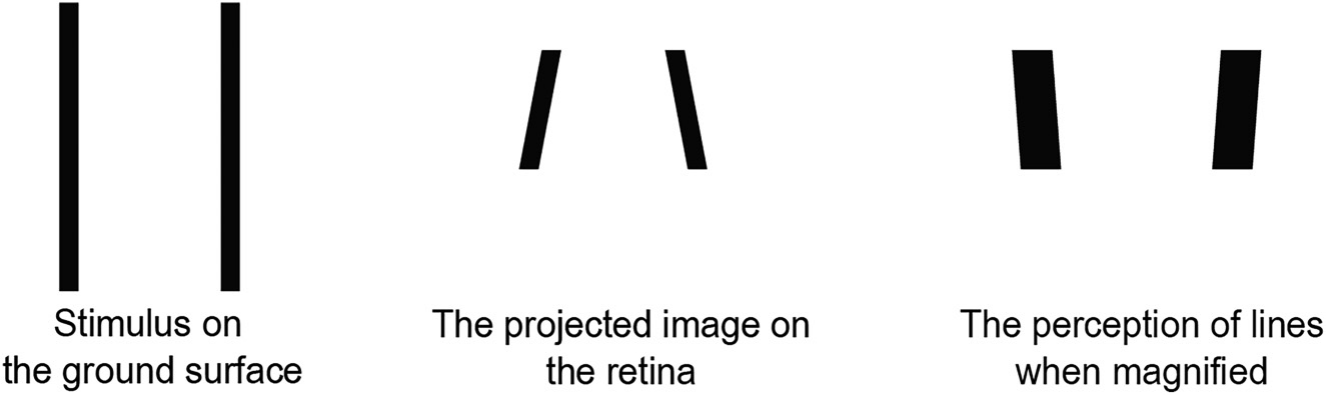
The example of the magnification illusion. Left: The stimulus on the ground surface. Middle: The projected image on the retina. Right: The perception of the same stimulus when magnified, as reported by Eltenton (1976).

**Figure 2.**
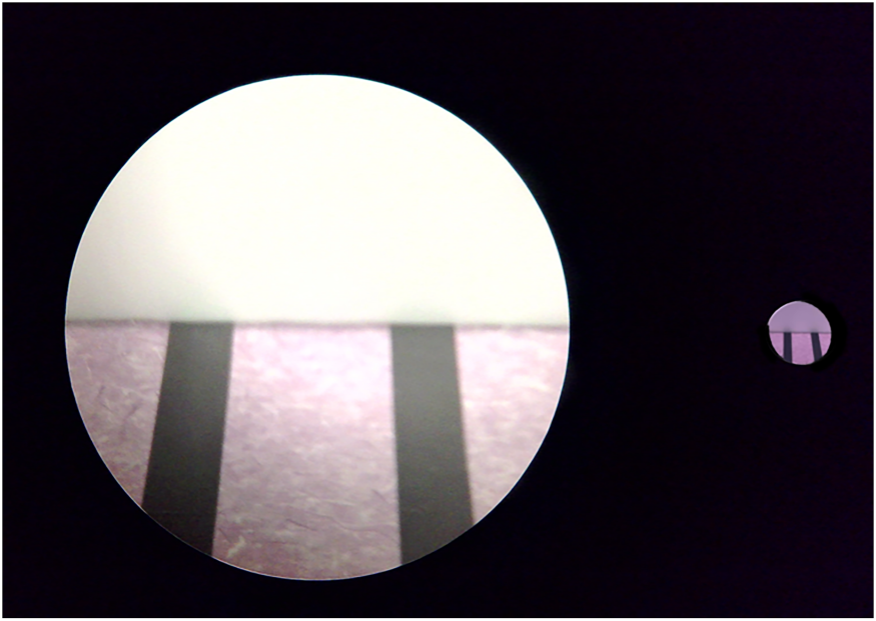
Images of the parallel lines stimulus in the ground setting of the experiment. The views are centered on the far edge of the stimuli. Left: The image was taken with a camera lens attached to binoculars. Right: The image was taken with the camera lens at the end of the paper cylinder tube.

When we look at two parallel lines on a floor running from the foot to infinity, the lines appear closer or converge into the distance. This linear perspective effect helps the visual system determine depth and spatial orientation. However, when one looks at the two parallel lines with a pair of binoculars, the expected convergence appears reversed, making parallel lines seem to diverge, as shown in Figure 1. We refer to this perception as “Magnification illusion.” perce The first documented report of this perceptual distortion was by Eltenton (1976). Later, Tsuinashi (2013) observed a similar effect when viewing rectangular stimuli on the ground through binoculars. To the best of our knowledge, only these two papers have mentioned this phenomenon. The underlying mechanism of this illusion remains unclear, and previous explanations have attributed it to potential optical distortions introduced by binocular lenses. However, our experimental findings contradict this explanation. Specifically, we observed that the retinal image formed through binoculars is an accurate magnification of the real scene, as shown in the left and right of Figure 2. This suggests that the illusion is not a result of optical distortions but rather a perceptual phenomenon tied to how the visual system interprets magnified scenes.

**Figure 3.**
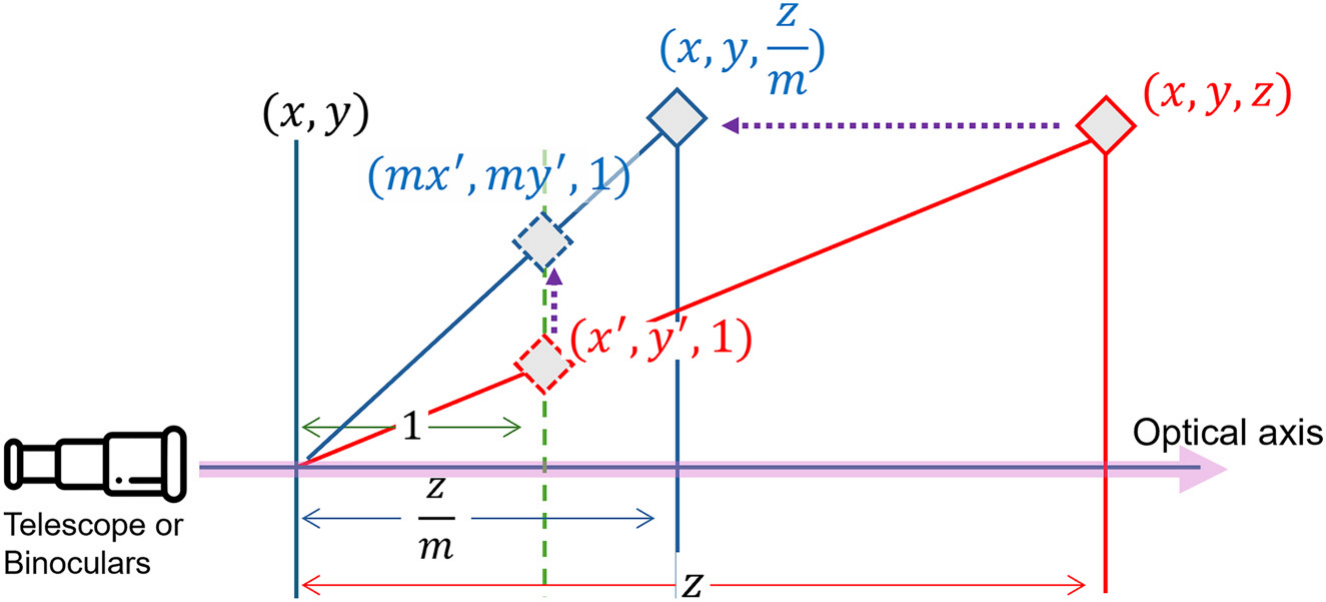
Perspective projection illustrating depth compression along the optical axis, z -axis, shown in pink. The green dashed line is the projection plane. The red diamond at (x,y,z) transforms under magnification into the blue diamond at (x,y,z/m) .

To explain this illusion, we proposed an alternative theory based on the depth compression Sripian et al. (2021, 2023). The interpretation of an angle between the lines in the three-dimensional (3D) scene is changed due to magnification, which may be related to depth compression. Objects seen through binoculars appear to have less depth, with regard to the binoculars’ optical axis, making them appear closer together than their actual distance. The perspective projection without magnification can be expressed with the following equation, given that a point (x,y,z) is projected to $\left(x^{\prime },y^{\prime },1\right)$ on z=1 plane as indicated in Figure 3 in red:

$$x^{\prime }=\frac{x}{z},\;y^{\prime }=\frac{y}{z}.$$

When the projected point is magnified with a factor of m , the projected point is moved to $\left(mx^{\prime },my^{\prime },1\right)$ as below:

(1) $$mx^{\prime }=m\frac{x}{z}=\frac{x}{z/m},\;my^{\prime }=m\frac{y}{z}=\frac{y}{z/m}.$$

Therefore, m -times magnification can be interpreted as if the original depth z is compressed to z/m , along the optical axis. In other words, objects in the scene appear closer than they actually are, as shown in blue, in Figure 3.

Binoculars consist of two parallel barrels that create aligned optical axes. This leads to depth compression along the optical axis that aligns with the midpoint of the barrels, which affects binocular disparity in a way that resembles depth compression. We perceive the objects as simply appearing closer when they are magnified.

**Figure 4.**
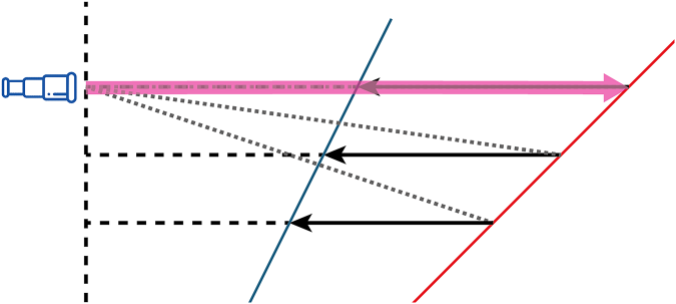
The concept of slant underestimation caused by depth compression along the optical axis: the plane (in red) appears closer as if the plane is less slanted (in blue).

Magnification alters depth perception, leading to slant angle underestimation due to depth compression along the optical axis, as illustrated in Figure 4. The relationship between retinal perspective, stimulus size, and slant perception has been extensively studied, with Freeman Jr (1966) demonstrating that both retinal perspective and stimulus size play critical roles in determining the absolute threshold for visual slant. Similarly, Braunstein and Payne (1969) found that relative slant judgments depend on form ratio and perspective cues. These findings indicate that retinal images serve as critical inputs for depth perception.

**Figure 5.**
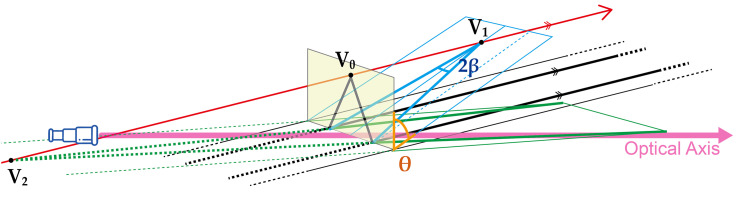
Illustration for multiple interpretations of one projected retinal image (yellow). One can interpret this image as being parallel lines placed on the ground (black lines), being converged and slanted upward (blue lines), or being diverged and slanted downward (green lines).

We assume that the depth compression might affect the perception of the slant angle and also the angles between two lines, which could cause the magnification illusion. The interpretation of the angles between two lines are changed according to the change of slant angle of the plane on which the lines lie. Figure 5 illustrates the change in the 3D interpretation of the two parallel lines on the ground, indicated in bold black lines. The parallel lines would converge at a vanishing point $V_{0}$ on the fronto-parallel plane (projected plane) in yellow. Suppose the distances to the parallel lines from the eye are equal; the projected parallel lines on the yellow plane would form an isosceles triangle. The projected isosceles triangle shown in Figure 5 can be reproduced by any stimuli like blue and green lines. The stimulus could be interpreted as any isosceles triangle with the same bottom, being parallel lines placed on the ground (black lines), being converged on a slanted ground (blue lines and $V_{1}$ as the apex), or being diverged on a slanted ground (green lines and $V_{2}$ as the apex). We define the interpreted angle between the projected plane and a plane of the isosceles triangle as *optical slant angle*, θ , while the half of its apex angle as *perspective angle*, β .

**Figure 6.**
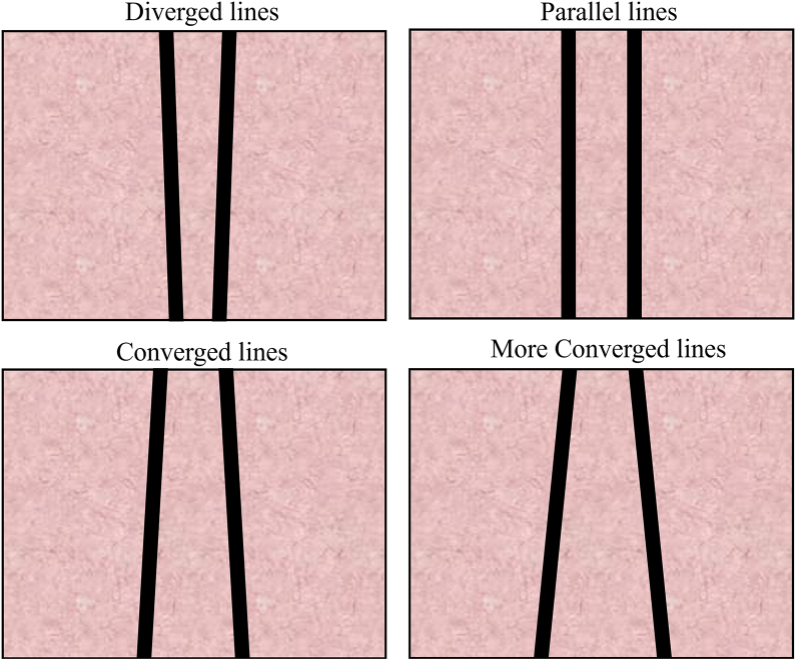
Four stimuli used in the experiment by Sripian et al. (2021) and Sripian et al. (2023).

We explored how depth compression influences the perception of two kinds of angles, the optical slant angle ( θ ) and the perspective angle ( β ). In this work, we redefine the magnification illusion as the change in the interpretation of these angles. We measured the observer’s perception of the stimulus using these two parameters ( β , θ ) in the experiment (Sripian et al., 2021, 2023). The stimuli used in the experiment consist of diverged lines, parallel lines, converged lines, and more converged lines, as shown in Figure 6. From the experiment, we found that the perspective angle, β , changes not only in the parallel lines but also in diverged lines and converged lines. Meanwhile, we also found that magnification also affects the interpretation of optical slant angle θ .

While setting up the experiment, we noticed that the perception of parallel lines placed in different locations, such as the ceiling or sidewall, was somewhat different from the perception on the ground when viewed through binoculars. This observation implies that changes in magnification might vary based on location, which could provide valuable cues to understand the magnification illusion. In this work, we explore the magnification illusion by examining the optical slant and perspective angles of two lines placed on the ground, sidewall, and ceiling.

### Distance Perception

The image of the external world on the retina is flat or 2D, but it is still possible to reproduce the 3D information with remarkable precision, even if perceived with a single eye. The visual system relies on depth cues to reconstruct 3D information from the 2D image projected on the retina. Cutting and Vishton (1995) adapted the plot from Nagata (1989) to form a just-discriminable depth thresholds as a function of the log of distance from the observer, from 0.5 to 5000 m, based on nine sources of information about the layout, which were convergence, accommodation, binocular disparity, motion, height, aerial, occlusion, relative size, and relative density. Based on the functions, the space around the observer could be divided into three types of space: personal space, action space, and vista space. However, their plot did not include important cues such as texture gradient, linear perspective, shading, and so on. For instance, the ground surface is vital for accurate egocentric distance judgment (Gibson, 1950; Sinai et al., 1998). Wu et al. (2004) showed that an observer judges the distance of an object by the sequential-surface-integration process. The surface integration process (He et al., 2004) is performed by integrating local patches of ground information into a global surface reference frame. This process reliably provides depth information, but it operates directionally, integrating cues primarily from near to far distances.

When a field of view (FOV) is restricted, the scene size could appear smaller (Creem-Regehr et al., 2005; Dolezal, 1982; Hagen et al., 1978). For example, the restricted FOV could corrupt spatial judgment because of its effect on reduced peripheral information (Dolezal, 1982). In other words, reduced FOV is found to compress perceived distance in the real environment experiments (Dolezal, 1982; Hagen et al., 1978), and also in the virtual environments (Loomis & Knapp, 2003; Thompson et al., 2004; Willemsen et al., 2008).

### Optical Slant Perception With Regard to Distance Perception in Various Locations

Over centuries, distance perception in human visual perception has been extensively investigated. Many studies reported that humans would compress egocentric distance, that is, perceived distance from an observer to an object, as much as 0.7 when observing the scene using the naked eye (Foley et al., 2004; Kelly et al., 2004). This depth compression may cause an underestimation in optical slant (Kammann, 1967; Proffitt et al., 1995; Wagner, 1985).

Li and Durgin (2010) conducted an experiment to investigate how perceived optical slant varies with different physical slants. Their methodology employed an innovative approach, using implicit judgments of optical slant based on an apparent isosceles right triangle on the eye-level surface. This method provided a novel way to assess slant perception without relying on explicit judgments. The results of their study closely aligned with the logarithmic function of viewing distance proposed by Bridgeman and Hoover (2008). They also introduced the angular expansion theory, which suggests an underlying gain for a perceived optical slant of ∼ 1.5 at each viewing distance. This finding is consistent with observations made by Durgin and Li (2011), where perceptual scale expansion was proposed as an efficient angular coding strategy for locomotor space.

Building upon their initial work, Li and Durgin (2012) later compared the angular expansion theory with the intrinsic bias hypothesis, providing additional insights into the mechanisms underlying slant underestimation. These findings collectively suggest that perceived optical slant is subject to systematic biases influenced by factors such as depth compression and binocular disparity information.

Slant perception is believed to be affected by different environmental surfaces. The ground theory proposed by Gibson (1950) suggested that there is visual anisotropy in slant perception and that surface continuity is important for organizing 3D scenes (Epstein & Park, 1964). Bian et al. (2005, 2006) found that human visual perception relies on the optical contact information provided by the ground, not by the ceiling, to organize 3D scenes. In their later work (Bian & Andersen, 2011), they also found that visual space perception when viewing a ground surface is less compressed than when viewing a ceiling surface. This suggests the unique role of the ground surface in the perceptual organization of 3D scenes. In the study of perceived slant from texture by Higashiyama and Yamazaki (2016), the pattern in which the direction of the gradient replicates that of the ground appears to be less slanted from the fronto-parallel plane, than the ceiling regardless of the head position.

### Magnification Illusion on the Ground

**Figure 7.**
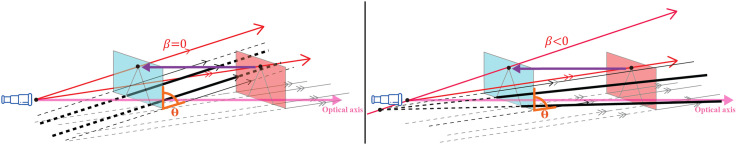
Two specific interpretations when viewing a scene through a telescope: left—parallel lines on a slanted plane, and right—diverging lines on a level plane. The fronto-parallel planes are colored red for naked-eye viewing conditions and blue for magnified viewing conditions.

According to Sripian et al. (2023), we hypothesized that the perception of perspective and optical slant angles would change when the scene is magnified. This perceptual shift could be attributed to changes in the 3D interpretation of lines during magnification. Figure 7 shows the two specific theoretical projection models of the possible changes in the interpretation when viewing the parallel lines under magnification.

To test this hypothesis, we analyze how the perceived perspective angle and the perceived optical slant deviate from the actual perspective and optical slant angles under different viewing conditions (magnification or naked eyes, under monocular vision or binocular vision) using multiple stimuli with different actual perspective angles placed on the ground. In the experiment, participants were asked to view different stimuli through binoculars (magnified viewing condition) using both eyes (binocular vision) and only one eye (monocular vision) and through paper cylinders (naked eyes) using both eyes (binocular vision) and only one eye (monocular vision). The results showed that the perspective angle is underestimated for all stimuli if magnified only when viewed with binocular vision. Meanwhile, the optical slant is underestimated even when viewed using the naked eye, and it is even more underestimated when magnified, regardless of binocular or monocular vision, when the stimuli are placed on the ground.

### Research Purpose and Hypothesis

In a previous experiment on the ground surface, (Sripian et al., 2023), we observed that the perspective angle β , as well as the optical slant angle θ , were somehow reduced when magnified. This finding supported the existence of the magnification illusion.

During that experiment, we noticed interesting variations in the interpretation change depending on the stimulus location. When viewed through the telescope, the stimulus in other locations appeared to slant towards the fronto-parallel plane, while the perspective angle remained unchanged, indicating a decrease in the optical slant angle only. The difference of the stimulus location merely causes the 2D rotation in the retinal image. We hypothesize that changes in the location of the stimulus, particularly due to rotations within the 2D retinal image plane, will influence the 3D interpretation of β and θ . Our primary hypothesis is that magnification illusion is caused by differences in 3D interpretation, like the left and right interpretations in Figure 7.

The interpretation change could arise from the nature of the surfaces themselves. The ceiling and sidewall are typically perceived as man-made structures, whereas the ground is a natural surface. Thus, the ground may be seen as a unique case that is different from the sidewalls and ceiling. On the other hand, differences in interpretation may be caused by binocular disparity. Research by Backus et al. (1999), Gillam and Lawergren (1983), Howard and Rogers (1996), Li and Durgin (2013), and Rogers and Bradshaw (1993) indicates that vertical and horizontal structures lead to variations in perception. While the standard terminology in vision science literature (Gillam & Lawergren, 1983; Howard & Rogers, 1996) distinguishes between slant (orientation about a vertical axis) and inclination (orientation about a horizontal axis), for reader convenience and consistency throughout this manuscript, we use the unified term ‘‘slant angle, θ ,” to refer to both types of surface orientations. The relationship between vertical disparity and horizontal disparity components becomes particularly relevant for sidewall orientations, where these binocular depth cues interact differently compared to ground and ceiling surfaces. In this context, sidewalls should be distinctly perceived from the ground and ceiling only when viewed with binocular vision. We want clarify the details of perception change in the location, which may lead us to understand the mechanism of the magnification illusion.

## Methods

In this study, our primary objective was to investigate how perspective angle and optical slant angle are perceived in different locations. The stimulus was placed on various surfaces, including the ground, ceiling, and sidewall, and participants’ perceptions were measured accordingly.

### Participants

Twenty-three students participated in the experiment, including 16 males and seven females. The average age of the participants was 24.5 years, with a standard deviation of 4.76 years. The research was conducted with the approval and compliance with the regulations of the Institutional Review Board of Shibaura Institute of Technology. Prior to the experiment, all participants provided informed consent, indicating their willingness to participate. The participants were not given any information about the study’s specific objectives. All participants had either normal vision or wore corrective lenses to ensure normal vision. To assess their stereo acuity, four random-dot stereograms were presented, and all participants were able to identify all the images displayed correctly.

### Stimulus

Similar to our previous experiment described by Sripian et al. (2023), we utilized four stimuli in this study. Figure 6 shows the four stimuli employed in the experiment: diverged lines, parallel lines, converged lines, and more converged lines, with physical perspective angles of −1∘ , 0∘ , 1.4∘ , and 3.75∘ , respectively. Each stimulus had dimensions of 91 cm × 60.5 cm. To enhance the plane’s visibility, bold black lines were drawn on a plate with a light pink textured background. We utilized a 1/f natural-noise texture, considered less effective in discriminating slant (Rosas et al., 2004). The texture has the mean luminance of 39.64 $cd/m^{2}$ , and 2.77 $cd/m^{2}$ for the lines, measured by Konica Minolta CS-200 chroma meter. The Weber contrast between the texture and the black lines stands at 13.31, indicating that it is quite easy to distinguish the black lines.

### Apparatus

**Figure 8.**
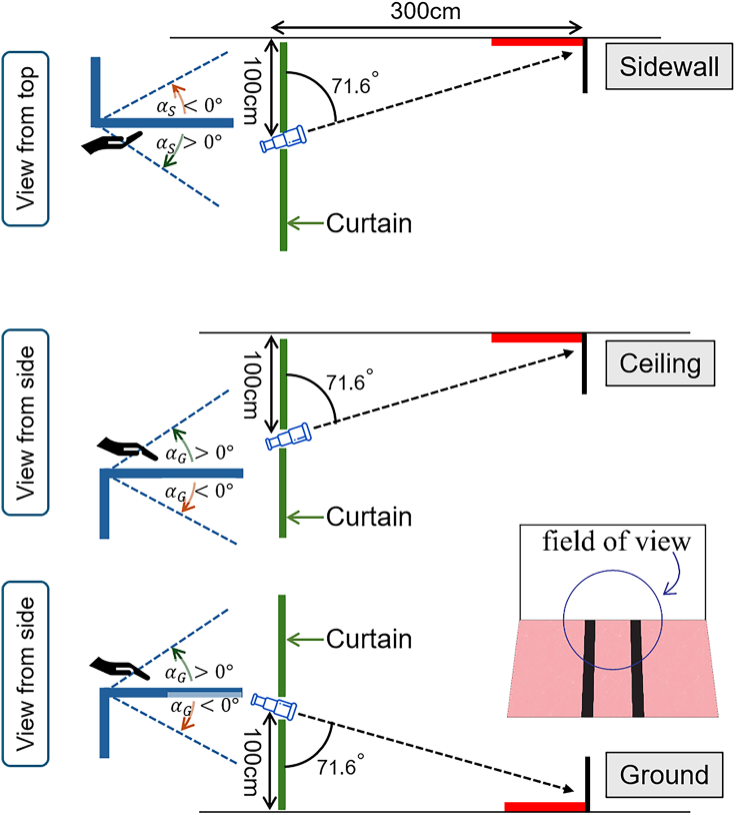
The experimental setup for the three locations.

In this study, we examined the perception of stimuli in three different locations: ground, sidewall, and ceiling. To set up the experiment, we employed specific apparatus and arrangements for each location. For the stimulus placed on the ground, we directly positioned it on the floor of the experiment room. For the stimuli on the ceiling and the sidewall, we designed stimulus holders that securely held the stimuli in place, parallel to the actual ceiling and sidewall surfaces. The slant of the stimuli remained constant throughout each location. Figure 8 illustrates the experimental room setup for all three stimulus locations.

To ensure that participants could only view the experimental setup through either binoculars or paper cylinders, the experiment area was covered by a curtain, leaving only a hole in the middle for the binoculars or paper cylinders to fit through. We used Olympus 8 × 21 RCII binoculars with 8 × magnification and a 21-mm objective lens for the binocular viewing condition. The paper cylinder has a diameter of 4 cm and a length of ∼ 30 cm. Therefore, the participant only saw the stimulus through a limited field of view. The FOV in magnification condition is 60.8∘ while FOV for paper cylinder is 7.6∘ .

Each set of viewing tools was mounted on its respective tripod, positioned 300 cm away from the far edge of the stimulus, and set at a height of 100 cm from the stimulus, as shown in Figure 8. Therefore, the optical axis is inclined at an angle of $71.6(=\tan^{-1}\;(100/300))\circ$ . The simulated FOV in the ground location is represented by the overlaid circle, indicating the restricted visual information available to the participant. While Figure 7 depicts isosceles triangles as a geometric interpretation of the vanishing points based on retinal projection, these vanishing points were not visible to the participants at all. The actual photos for magnified and naked eye conditions taken from the ground location are shown in Figure 2.

**Figure 9.**
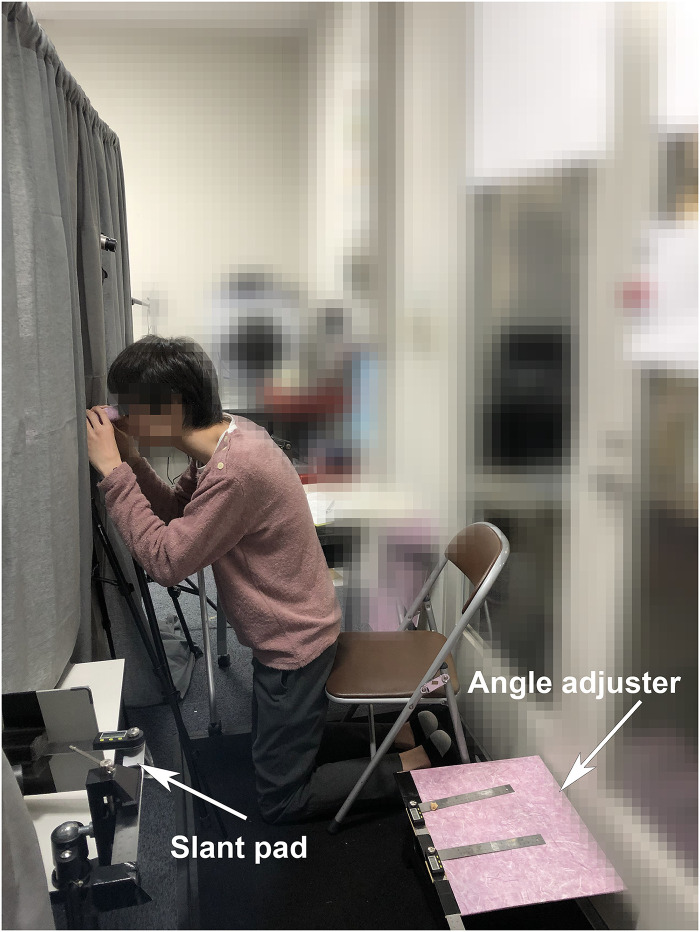
The photo from the experiment. The participant was looking at the stimulus placed on the sidewall, therefore the slant pad is designed to replicate the direction accordingly. The angle adjuster is placed on the small table near the participant.

**Figure 10.**
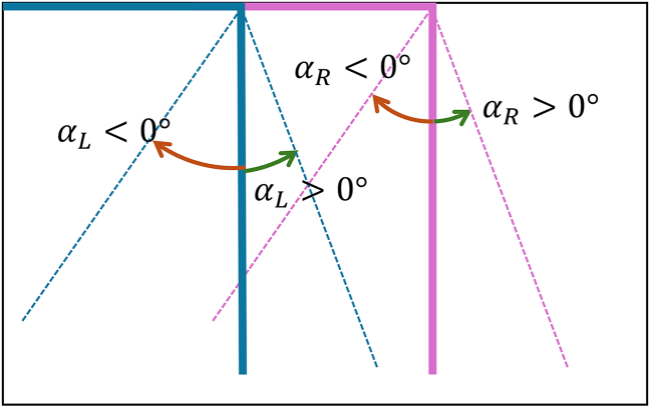
The design of angle adjuster.

Figure 9 shows a photo of the experiment setup. We developed customized tools to facilitate the measurement and replication of perceived angles. For the perspective angle, we created an angle adjuster using two digital angle finders, measuring $\alpha_{L}$ and $\alpha_{R}$ , as shown in Figure 10. The participant was asked to adjust the two digital angle finders to make them parallel to the two lines of the stimulus. Both digital angle finders are set to 0∘ when the rulers are parallel to the optical axis. This angle adjuster was positioned close to the participant when viewing the stimulus.

To measure the slant angle, we designed a slant pad inspired by a palm board (Proffitt et al., 1995) and attached a digital angle finder to it. For the ground and ceiling stimuli, the slant pad which measures $\alpha_{G}$ , was fixed to the table to replicate the level ground or level ceiling when the digital angle finder is set to 0∘ . The configuration of the slant pad and its adjustments by the participant are illustrated in Figure 8. The participant had to adjust the slant pad upward or downward until it was parallel with their perceived slant of the ground or ceiling stimulus. Although the slant pad can be adjusted from either above or below to match the angle of the ground or ceiling, all participants chose to adjust the slant pad using a downward-facing palm to replicate the slant of the ground or the ceiling stimulus. For the sidewall stimuli, a separate slant pad, measuring $\alpha_{S}$ , was positioned vertically on the table to replicate an artificial vertical wall. Participants adjusted this pad leftward or rightward until it was parallel with their interpretation of the sidewall stimulus slant. The digital angle finder was set to 0∘ when this pad was parallel to an artificial sidewall.

### Design

There are four types of conditions in the experiment;

- **LO** (location): ground, sidewall, and ceiling
- **NE** (number of eyes used): monocular vision (dominant eye) and binocular vision
- **ST** (stimulus): diverged, parallel, converged, and more converged
- **VC** (viewing condition): magnified and naked eye.

Each participant completed trials across all experimental conditions in a controlled sequence described in Experimental Sequence 1. The overall sequence of the experiment was defined for ease of experimental execution.

**Table:** Experimental Sequence 1: Stimulus presentation

**foreach** *LO in* {*Ground, Ceiling, Sidewall*} **do**
**foreach** *VC in* {*Magnified, Naked Eye*} **do**
**foreach** *NE in* {*Monocular, Binocular*} **do**
Randomize Stimuli Order (1-4);
**foreach** *ST in Randomized Order* **do**
Present Stimulus;
Record Angles;
**end**
**end**
**end**
**end**

Participants viewed four stimuli presented in a fully randomized order to avoid biases. For each stimulus, the participant was asked to estimate the stimulus’s perspective and optical slant angles. The participants first looked at the stimulus and then adjusted the digital angle finders until the angle adjuster and slant pad replicated their answers. After reaching a satisfactory alignment, participants directly recorded the angles $\alpha_{R}$ , $\alpha_{L}$ , $\alpha_{G}$ , and $\alpha_{S}$ from the digital display attached to the angle adjuster and the slant pad. This was necessary because the experimenter remained positioned behind a curtain, unable to view the digital angle finders. While repeated presentations of identical stimuli would have better assessed measurement consistency, this would have significantly increased experiment duration and participant burden, potentially introducing fatigue effects that could compromise data quality. Therefore, as a methodological compromise, we randomized stimulus presentation order across participants to minimize systematic carryover effects while maintaining experimental feasibility.

For the perspective angle, β , participants adjusted two rulers on the angle adjuster to make each ruler parallel to the corresponding stimulus line as viewed through the binoculars or paper cylinders. The β value was calculated as the half of the difference of these two numbers, that is, $\beta =\alpha_{R}-\alpha_{L}/2$ , where $\alpha_{L}$ is the measured angle from the left adjuster, and $\alpha_{R}$ is the measured angle from the right adjuster.

For the optical slant angle, θ , participants adjusted the slant pad to make it parallel to the perceived orientation of the stimulus surface as viewed through the binoculars or paper cylinders. The θ value for the ground location was calculated as: $\theta =71.6\circ -\alpha_{G}$ , while for the ceiling location the θ was calculated using the reverse formula: $\theta =\alpha_{G}-71.6\circ$ , where $\alpha_{G}$ is the measured angle from the digital angle finder attached to the slant pad. For the sidewall, the value of θ is calculated by $\theta =71.6\circ -\alpha_{S}$ , where $\alpha_{S}$ is the measured angle from the digital angle finder attached to the sidewall slant pad.

After finishing the recording, the participant was instructed to sit on a chair until the experimenter had finished changing the stimulus. The participant was not allowed to see the stimulus during the changing time. In other words, he/she was permitted to observe their surroundings on his/her side of the curtain. The experimenter stayed with the participant the whole time. The participant was not given any feedback regarding their performances.

## Results and Discussion

This section presents our findings on the changes in perspective angle ( β ) and optical slant angle ( θ ) under various experimental conditions. Our analysis focuses on how these angles are affected by viewing condition (VC: magnified vs. naked eye), location (LO: ground, sidewall, and ceiling), number of eyes used (NE: monocular vs. binocular), and stimulus type (ST: diverged lines, parallel lines, converged lines, and more converged lines).

We conducted a four-way repeated measures ANOVA to comprehensively analyze the complex interactions between these multiple factors. This section summarizes the main findings and their implications. For detailed results, including ANOVA outcomes, pairwise comparisons, and interaction effects, please refer to the Appendix. We will first look at the effects on β and θ individually and then analyze how they are related through a proposed computational model.

Our primary hypothesis was that changes in stimulus location would influence the changes in β and θ . Therefore, we pay special attention to how viewing conditions affect these angles across different locations. The key findings from individual analysis of perspective angle and optical slant angles are described below.

### Analysis on Perspective Angle

**Figure 11.**
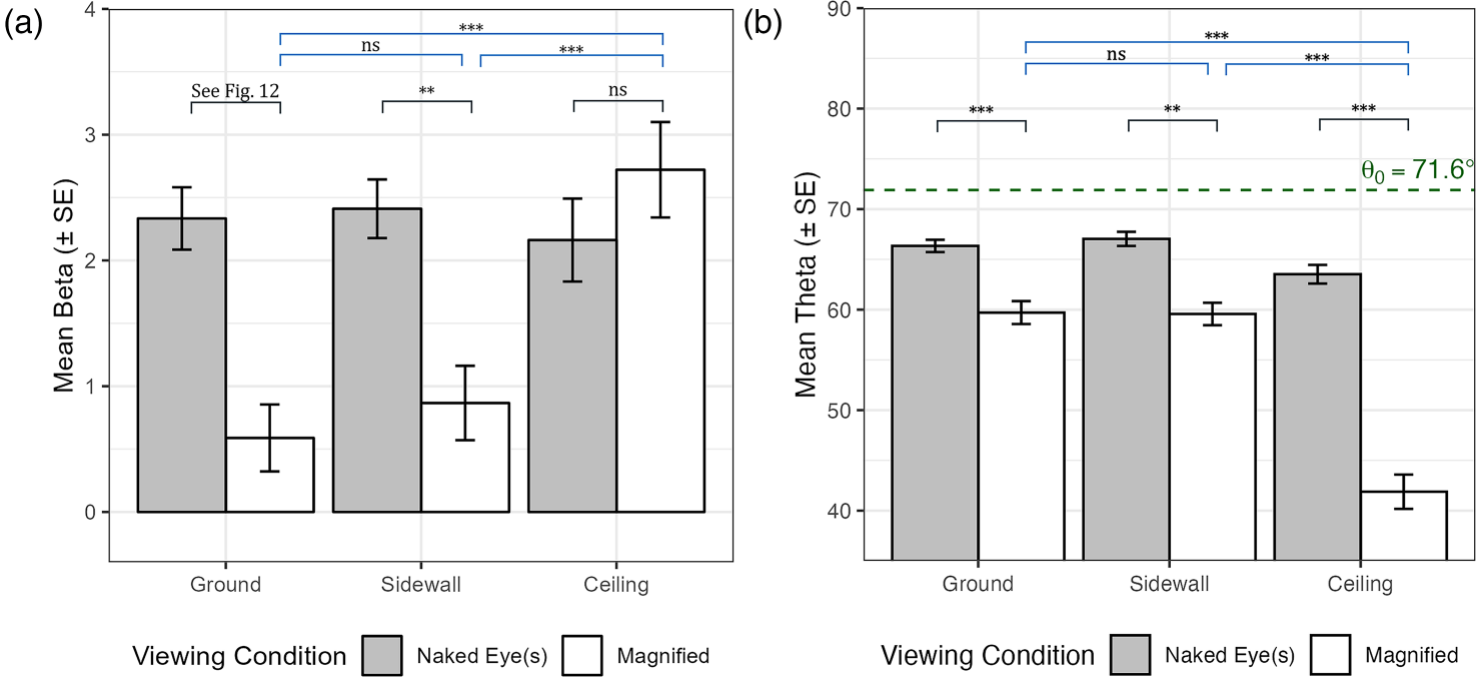
Comparison of perspective angle β and optical slant angle θ across different locations. The plot includes standard error bars, with significant differences indicated by pairwise comparisons shown above the bars. (a) Average perspective angle β for different locations irrespective of the NE and ST. (b) Average optical slant angle θ for different locations irrespective of NE and ST. NE = number of eyes used; ST = stimulus type.

**Figure 12.**
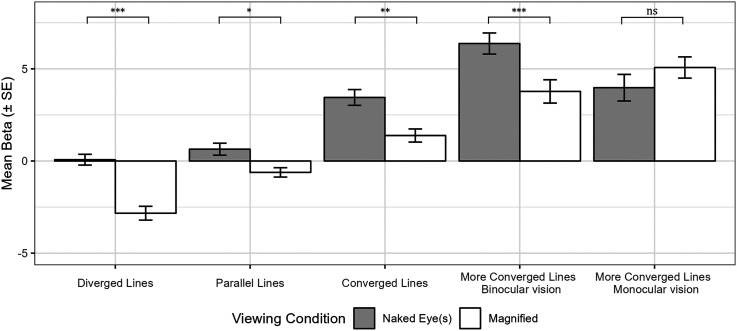
Average perspective angle β for the ground location plotted with standard error bars. The bar charts are organized based on ST, regardless of NE. Due to a significant interaction in the statistical results, the more converged lines are further separated by NE. Significant differences from pairwise comparisons are indicated above the bars (see the Appendix for more details). NE = number of eyes used; ST = stimulus type.

The analysis of perspective angle β focused on the effect of VC and the influence of LO. We present the results in Figure 11(a), where the black lines represent the comparisons between VCs. For the sidewall and ceiling locations, no interactions were found among the VC, NE, and ST, allowing us to analyze the effects of VC while ignoring NE and ST. The sidewall location showed a significant difference in β between VCs. The ceiling condition did not exhibit significant differences between VCs. A detailed breakdown is required only for the ground location where a significant three-way interaction was observed. The comparisons for the ground location are shown in Figure 12. We found significant differences in β when magnified in all cases except for more converged lines viewed using monocular vision.

For the comparison among LOs, we found no significant main effect of LO on β in the naked-eye condition. However, under magnification (blue comparison lines of Figure 11(a)), significant differences were observed in the ground-ceiling and sidewall-ceiling comparisons but no significant differences for the ground-sidewall comparison. This result shows that the interpretation of perspective angle is significantly unique if the stimulus is placed on the ceiling when magnified.

### Analysis on Optical Slant Angle

The analysis of optical slant angle θ focused on the effects of VC and LO. No significant interactions were found among VC, NE, and ST for optical slant angle ( θ ), allowing us to analyze the effects of VC while disregarding NE and ST across all three locations. At each location, we found statistically significant differences between VCs (black comparison lines in Figure 11(b)), demonstrating a strong effect of magnification on optical slant perception.

In terms of comparisons among LO, there was a significant interaction effect of LO × VC. Pairwise comparisons revealed significant differences only within the magnified viewing condition (blue comparison lines in Figure 11(b)), where significant differences appeared in the ground-ceiling and sidewall-ceiling comparisons, similar to the results observed with the perspective angle.

In our experiment, the slants of all the stimuli are the same (always 71.6∘ ) as shown in Figure 8. From the result shown in Figure 11(b), it can be seen that even without magnification, the slant seems to be underestimated. When magnified, the slant is underestimate even more, and the most when the stimuli is placed on the ceiling.

The perception of optical slant is influenced by the reference frame used to define zero slant. Kammann (1967) and Proffitt et al. (1995) measured geographical slant rather than optical slant, with Proffitt emphasizing an overestimation of slant when zero was defined relative to the horizontal plane. In contrast, when optical slant is defined with fronto-parallel as zero, it is typically underestimated, as reported in studies such as Wagner (1985). Additionally, Li and Durgin (2013) demonstrated that perceived optical slant exhibits a nonlinear bias and interacts with binocular disparity information. Consistent with these findings, our experiment also revealed an underestimation of optical slant when defined relative to a fronto-parallel reference frame.

The analysis of perspective angle ( β ) and optical slant angle ( θ ) shows the significant effect of magnification in most cases. Importantly, changes in location, which correspond to rotations within the 2D retinal image plane, result in perception differences, indicating a shift in 3D interpretation. In our results, we observed distinct effects at different locations: the ceiling showed a significant reduction in θ under magnification, while the ground and sidewall locations responded similarly but less prominently.

Our prior expectation was either that the ground would be distinct from other locations because of its nature being a natural surface or that the sidewall would be distinct due to the relationship between vertical disparity and horizontal disparity components in binocular vision. However, the observation from the experiment result shows that the ceiling was otherwise, distinct from the other location. Further experiments will be necessary to better understand the nature of this discrepancy.

### Relationship of the Perspective Angle and Optical Slant Angle Regarding Magnification

While the previous section analyzed β and θ separately, it is important to recognize that these two parameters are interconnected and hold a relationship with the magnification factor. To explore this relationship, we propose a mathematical model that links β , θ , and the magnification factor.

**Figure 13.**
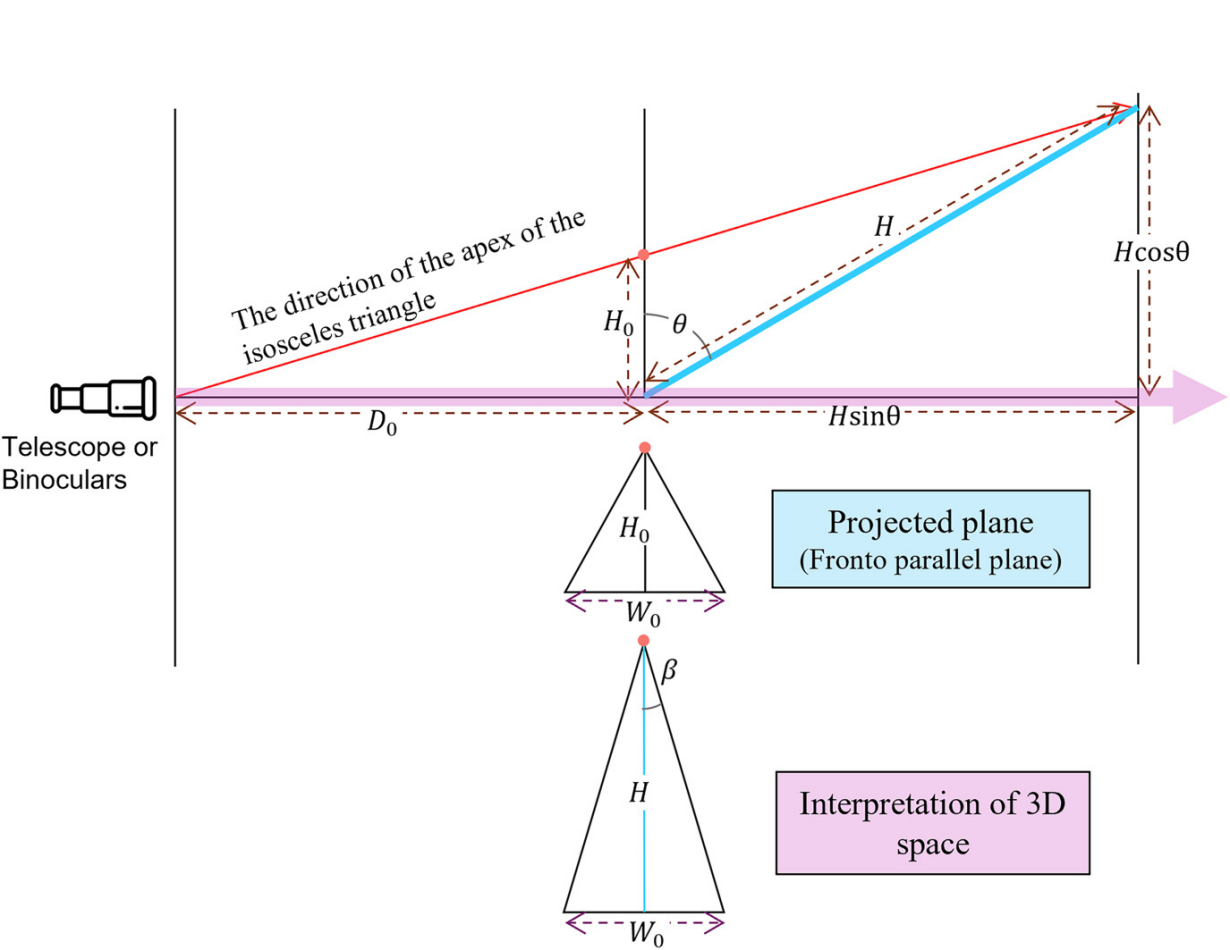
The relationship of the interpretations of perspective angle β and optical slant angle θ with regard to $D_{0}$ .

Let us discuss the relationship between the perspective angle β and the optical slant angle θ of the 3D interpretation with the same projected image. The projected image is an isosceles triangle whose bottom width and height are $W_{0}$ and $H_{0}$ , respectively, as shown in the middle of Figure 13. From the bottom figure, the following equation between β and the height of the interpreted isosceles triangle H is obtained:

(2) $$\tan\;\beta =\frac{W_{0}}{2H}.$$

From the top figure, we can obtain the relation between θ and H as below:

(3) $$\frac{D_{0}}{H_{0}}=\frac{D_{0}+H\sin\;\theta }{H\cos\;\theta },$$

where $D_{0}$ indicates the distance from the eyes to the projected plane. By removing H from Equations (2) and (3), we obtain the equation of the perspective angle β and optical slant angle θ with regard to $D_{0}$ as follows:

$$\beta =\tan^{-1}\;\frac{W_{0}\cos\;\theta \left(D_{0}-H_{0}\tan\;\theta \right)}{2D_{0}H_{0}},$$

**Figure 14.**
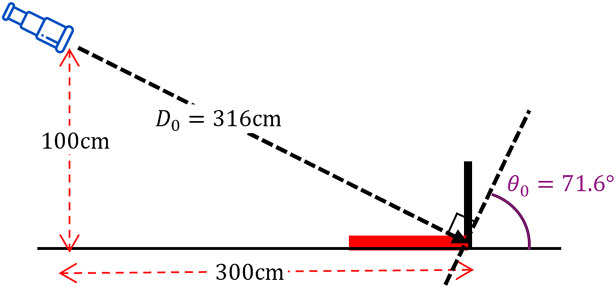
The setup of telescope viewing the parallel lines stimulus (shown in red) placed on a level ground, at the height of 100 cm and the horizontal distance from the stimulus of 300 cm, resulting in the distance from the eyes to the stimulus, $D_{0}=316$ cm and the actual slant angle $\theta_{0}=71.6^{\circ }$ .

$H_{0}$ can be obtained with the actual 3D scene configurations, given as $D_{0}$ , $W_{0}$ , $\theta_{0}$ , and $\beta_{0}$ . For example, when we observe parallel lines placed on the ground, as shown in Figure 14, the actual distance $D_{0}$ is $316(=\sqrt{300^{2}+100^{2}})$ cm, and the bottom width of the stimulus $W_{0}$ is 17 cm, the actual optical slant angle $\theta_{0}$ is $71.6(=\tan^{-1}\;(100/300))\circ$ , and the actual perspective angle $\beta_{0}=0\circ$ .

When a scene is enlarged, the height and the width of the isosceles triangle, $H_{0}$ and $W_{0}$ , would be magnified by m , which is equivalent to compressing the $D_{0}$ by 1/m , as shown in the following equation, which was also indicated in Equation (1).

(4) $$\begin{array}{ccc} \beta & = & \tan^{-1}\;\frac{(mW_{0})\cos\;\theta \left(D_{0}-(mH_{0})\tan\;\theta \right)}{2D_{0}(mH_{0})}\\ & = & \tan^{-1}\;\frac{W_{0}\cos\;\theta \left(\frac{D_{0}}{m}-H_{0}\tan\;\theta \right)}{2\frac{D_{0}}{m}H_{0}}. \end{array}$$

**Figure 15.**
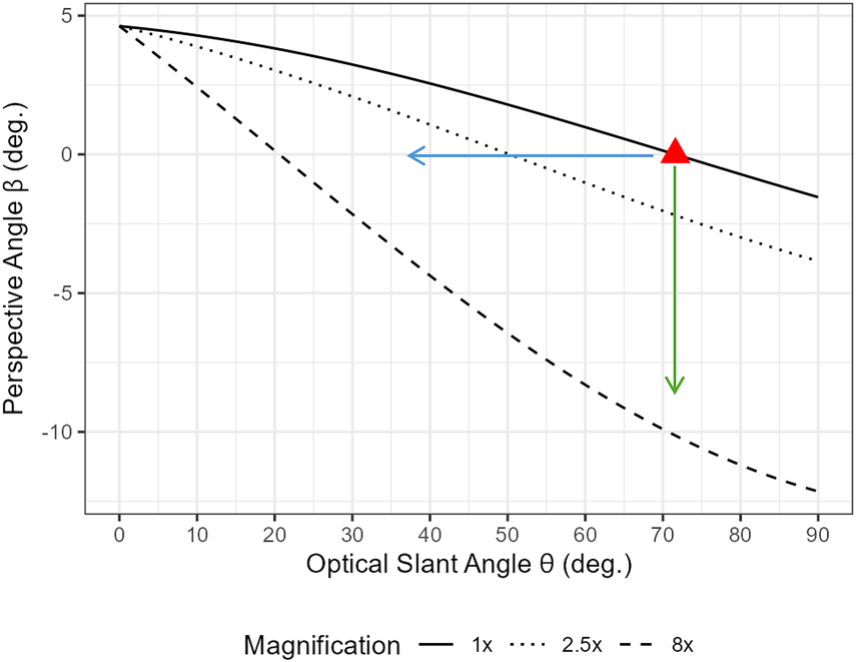
The relationship between the interpretations of the perspective angle β and the optical slant angle θ varies with the magnification factor, under the conditions depicted in Figure 14.

From the equation, it is possible to plot the relationship between the interpretation of β and θ with regard to the magnification factor m . Suppose the magnification factor is 1 × ; the relationship is depicted with a solid-line plot in Figure 15. A red triangle indicates the actual optical slant angle $\theta_{0}=71.6\circ$ and the actual perspective angle $\beta_{0}=0\circ$ for the parallel line.

Meanwhile, when a scene is magnified by factors of 2.5 × and 8 × , the relationships between β and θ can be plotted using dotted and dashed lines, respectively. Notably, as the magnification factor m increases, both the perspective angle β and the optical slant angle θ tend to decrease, resulting in a shift of the lines from the upper right to the lower left of the plot.

Assuming the hypothesis of changes in 3D interpretation, we identify two specific cases of the possible interpretations of the perspective angle and the optical slant angle, as illustrated in Figure 7. In the context of this hypothesis, Figure 15 represents the following two characteristic examples, depicted as arrows on the graph.

1. The lines remain parallel while they are placed on a slanted plane (Figure 7 left).
2. The lines are placed on a level plane, causing them to no longer appear parallel (Figure 7 right).

The first interpretation corresponds to the blue leftward arrow in Figure 15, where β is fixed. Meanwhile, the second interpretation is depicted by the green downward arrow in Figure 15, where θ is fixed.

### Changes of β and θ When Magnified: Combined Analysis

**Figure 16.**
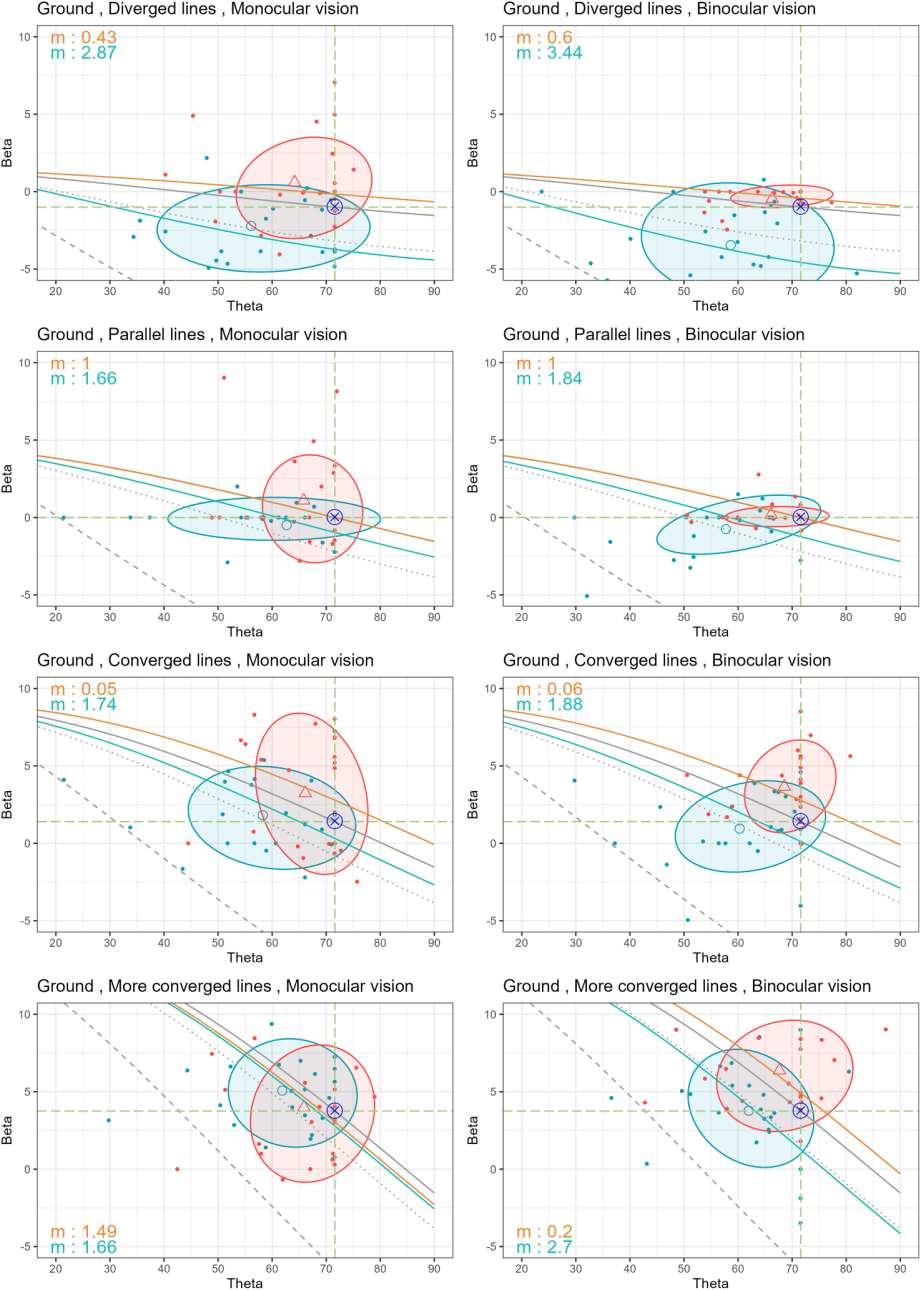
Observation results of β and θ under monocular vision (left graphs) and binocular vision (right graphs) for each stimulus placed on the ground. Orange and blue dots indicate each participant’s response in the naked-eye and magnified conditions, respectively. Orange triangles and blue circles represent the average β and θ values for the naked-eye and magnified conditions, with 68% confidence ellipses, respectively. The ⊗ symbol marks the ground truth. The gray lines represent the functions of β and θ at different magnification factors: 1 × (solid), 2.5 × (dotted), and 8 × (dashed), while solid orange and blue lines show fitted models for the naked-eye and magnified conditions, respectively, with the best-fitted magnification factor m shown on the graphs.

**Figure 17.**
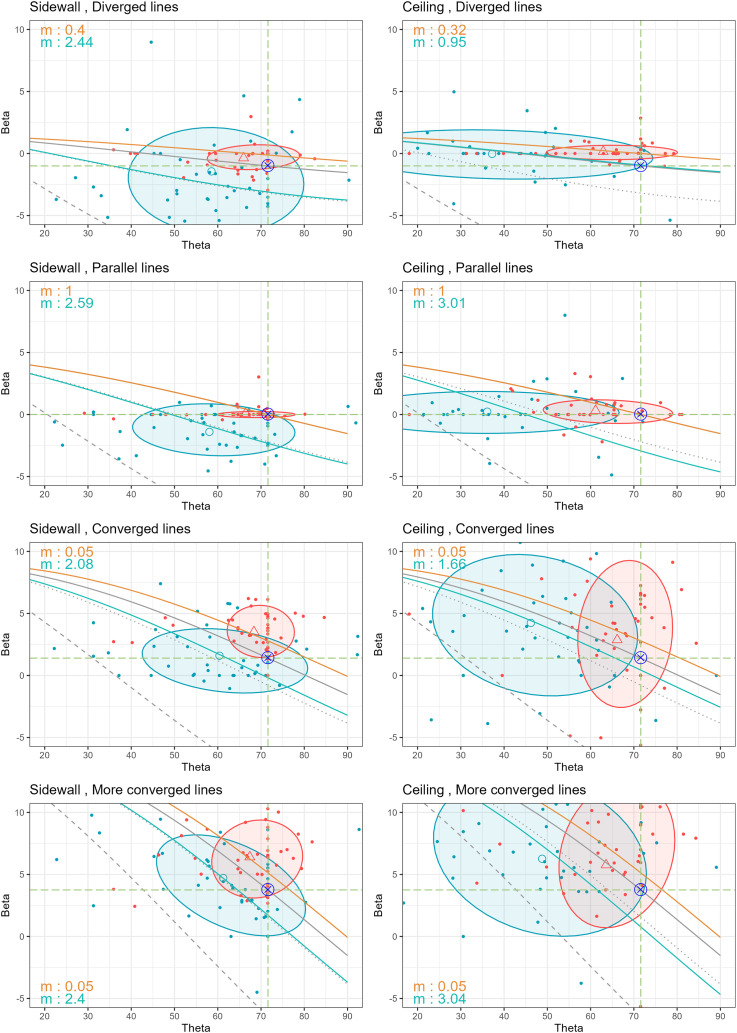
Observation results of β and θ for each stimulus placed on the sidewall (left graphs) and ceiling (right graphs), regardless of the number of eyes used. Orange and blue dots indicate each participant’s response in the naked-eye and magnified conditions, respectively. Orange triangles and blue circles represent the average β and θ values for the naked eye and magnified conditions, with 68% confidence ellipses, respectively. The ⊗ symbol marks the ground truth. The gray lines represent the functions of β and θ at different magnification factors: 1 × (solid), 2.5 × (dotted), and 8 × (dashed), while solid orange and blue lines show fitted models for the naked-eye and magnified conditions, respectively, with the best-fitted magnification factor m shown on the graphs.

The observation results of β and θ at different locations (ground, sidewall, and ceiling) are shown in Figures 16 and 17. Only the ground location results are separated into monocular and binocular vision in Figure 16, as indicated by the results of the statistical analysis of the perspective angle discussed in the Appendix. Each participant’s response is plotted using colored dots, where an orange dot represents naked-eye conditions and a blue dot represents the magnified condition. Dashed green lines indicate the actual values of $\beta_{0}$ and $\theta_{0}$ , marked by ⨂ at their intersection. Grey lines illustrate the relationship between β and θ for different magnification factors. The line styles represent magnification ratios similar to those in Figure 15. While the mathematical model introduced in the previous section establishes a theoretical relationship between the perspective angle β and optical slant angle θ , it is important to note that participants’ perceptual reports may not strictly adhere to this theoretical curve. Therefore, we analyze β and θ as related but distinct perceptual measures that reflect the complexity of 3D spatial interpretation under magnification.

The average values of β and θ under naked-eye conditions are represented by orange triangles ( △ ) in each graph, along with their 68% confidence ellipses. Similarly, blue circles ( ◯ ) represent the average values under magnified conditions with the corresponding confidence ellipses. In both ground and sidewall locations (with the exception of the more converged line in the ground location), the change from orange triangles to blue circles moves toward the lower left of the plot. This indicates a reduction in both angles when magnified. In the ceiling location, however, the shift caused by magnification primarily occurred in the horizontal direction (to the left) rather than vertically. Especially for the converged and more converged lines, the change is slightly upward. This resulted in a unique pattern, with θ values showing a significant reduction while β remains almost unchanged, compared to both the ground and sidewall.

To verify whether our observed data aligns with the hypothesis of changes in 3D interpretation, we fitted the data to the relationships derived from this hypothesis, optimizing the parameter m to match the β and θ values. The difference was quantified using a sum of squared errors (SSE) which calculates the total squared differences between the predicted and observed values of β . The optimization was carried out using the golden section search method. The search was performed over a predefined range of parameter value m∈[0.05,∞] , and the method ensures convergence to an optimal solution within a specified tolerance level of 0.0001.

We illustrate the best-fit magnifications for the naked-eye and magnified conditions using orange and blue solid lines in Figures 16 and 17, with the m values for each viewing condition indicated on the respective graphs. For the naked-eye condition (orange solid lines), the best-fit magnifications are generally much smaller than the actual magnification factor of 1 × . In some cases, particularly for the naked-eye condition in converged lines of the sidewall and ceiling location in Figure 17, the best-fit magnifications approach 0.05, which is constrained by the predefined range of parameter m . On the other hand, for the magnified condition (blue solid lines), the best-fit magnifications are closer to 2.5 × , lower than the actual binocular specification of 8 × . When comparing the magnification factor obtained from the naked-eye viewing condition with that of the magnified viewing condition, it consistently shows that magnification factor increase when magnified.

It is to be noted that only in the case of the parallel lines viewed under the naked-eye condition did the best-fit magnification align perfectly with the actual 1 × , regardless of the location of the stimulus, and number of eyes, according to the second rows of Figures 16 and 17. This observation further emphasizes that parallel lines are unique to human visual perception. The brain appears to process parallel stimuli differently, which requires further investigation.

## Conclusion

This study explored the effects of magnification on the perception of perspective angle and optical slant angle for stimuli placed on the ground, sidewall, and ceiling. The results indicated that the perspective or optical slant angle is influenced by magnification, with varying degrees of effect depending on where the stimulus is positioned. Importantly, these differences in perception arise only from rotations within the 2D retinal image plane, which indicates that this illusion is caused by the change in 3D interpretation. We developed a calculation model based on the hypothesis of changes in 3D interpretation to describe the relationship between the perspective angle, optical slant angle, and magnification factor. By fitting the observed data to this model, we found that the data support the hypothesis, indicating that the magnification factor is larger when using binoculars compared to the naked-eye condition. This alignment between the model and data reinforces the idea that changes in 3D interpretation are involved.

We initially expected that if natural surface priors influenced perception, the ground location would show unique effects, whereas if binocular disparity played a key role, the sidewall would be distinct. Specifically, we hypothesized that the relationship between vertical disparity and horizontal disparity components in binocular vision would become particularly relevant for sidewall orientations, as outlined in our hypothesis. However, our results did not support either expectation. Instead, we found that the ceiling location exhibited a distinct response, suggesting a unique perceptual mechanism at play. Additionally, one notable exception was observed in the naked-eye condition for parallel lines, where the magnification factor accurately matched the actual value (1 × ), regardless of stimulus location or number of eyes used.

These findings could enhance our understanding of how magnification affects spatial perception and provide important insights for designing virtual reality (VR) and augmented reality (AR) systems, where depth and angle perceptions are critical. Yet, the underlying reasons for the ceiling’s unique perceptual response to magnification and the interpretation of parallel lines under the naked-eye condition remain unclear and require future investigation.

## Supplemental Material

sj-csv-1-ipe-10.1177_20416695251351412 - Supplemental material for Magnification effects on perspective angle and optical slant angle across locations Supplemental material, sj-csv-1-ipe-10.1177_20416695251351412 for Magnification effects on perspective angle and optical slant angle across locations by Peeraya Sripian, Takashi Ijiri, and Yasushi Yamaguchi in i-Perception

## Appendix

### Perspective Angle

We first conducted a four-way ANOVA, which revealed a significant four-way interaction. As a result, we proceeded with a three-way ANOVA, fixing LO, because our main concern is the difference between VCs. The results indicated a significant three-way interaction for ground, but not for sidewall or ceiling. Consequently, we conducted a two-way ANOVA for ground, fixing ST, and found the significant interaction only for more converged lines.

We also conducted a three-way ANOVA, fixing VC, because our second concern is the difference between LOs. The results revealed a main effect for LO in the magnified condition, prompting us to perform pairwise analyses. Additionally, there was a three-way interaction only for the naked-eye condition. Therefore, we conducted separate two-way ANOVAs for binocular and monocular conditions. The results of these two-way ANOVAs showed only a main effect for the ST, but not for LO.

The full series of hierarchical ANOVA analyses, from four-way ANOVA down to 2-way ANOVAs, is summarized in Table 1. To improve clarity and reduce clutter, only results up to the level where significant interactions were found are included.

**Table 1.** Comprehensive Hierarchical ANOVA Results for Perspective Angle.

Analysis level	Effect	Sum of squares	*df*	Mean square	*F*-value	*p*-value
*Four-way ANOVA*						
	LO × VC × NE × ST	83.852	6	13.975	2.893	**.011**
*Three-way ANOVA (fixing LO)*						
Ground	VC × NE × ST	61.735	3	20.578	5.295	**.002**
Sidewall	VC	219.444	1	219.444	33.223	**<.001**
	NE	0.114	1	0.114	0.023	.882
	ST	2570.850	3	856.950	88.340	**<.001**
	VC × NE	4.663	1	4.663	1.081	.310
	VC × ST	8.916	3	2.972	0.536	.660
	NE × ST	2.172	3	0.724	0.209	.889
Ceiling	VC	28.735	1	28.735	1.465	.239
	NE	2.432	1	2.432	0.180	.676
	ST	2261.805	3	753.935	24.882	**<.001**
	VC × NE	5.912E-5	1	5.912E-5	0.000	.997
	VC × ST	49.861	3	16.620	1.777	.160
	NE × ST	20.820	3	6.940	0.880	.456
*Two-way ANOVA (fixing ST for ground)*						
Diverged lines	VC	193.865	1	193.865	28.739	**<.001**
	NE	30.073	1	30.073	11.681	**.002**
	VC × NE	0.178	1	0.178	0.056	.815
Parallel lines	VC	36.502	1	36.502	6.835	**.016**
	NE	7.895	1	7.895	2.418	.134
	VC × NE	2.528	1	2.528	1.178	.290
Converged lines	VC	98.149	1	98.149	13.701	**.001**
	NE	1.136	1	1.136	0.154	.699
	VC × NE	9.475	1	9.475	2.194	.153
More converged lines	VC × NE	78.579	1	78.579	20.730	**<.001**
*Three-way ANOVA (fixing VC)*						
Magnified	LO	494.521	2	247.261	8.339	**<.001**
	NE	10.192	1	10.192	1.024	.323
	ST	3909.904	3	1303.301	97.552	**<.001**
	LO × NE	32.433	2	16.216	1.875	.165
	LO × ST	78.731	6	13.122	1.130	.349
	NE × ST	9.776	3	3.259	0.477	.699
	LO × NE × ST	43.395	6	7.232	1.142	.342
Naked eye(s)	LO × NE × ST	65.952	6	10.992	3.061	**.008**
*Two-way ANOVA (fixing NE for naked eye(s))*						
Binocular, naked eye	LO	6.091	2	3.045	0.334	.718
	ST	1746.537	3	582.179	109.932	**<.001**
	LO × ST	27.498	6	4.583	1.068	.385
Monocular, naked eye	LO	4.492	2	2.246	0.140	.870
	ST	1227.118	3	409.039	44.801	**<.001**
	LO × ST	100.250	6	16.708	1.731	.118

Significant differences ( p<.05 ) are Bonferroni corrected.

ANOVA = analysis of variance; LO = location; VC = viewing condition; NE = number of eyes used; ST = stimulus type.

Accordingly, we perform pairwise comparisons among VCs and LOs for the cases where the main effects were found. Table 2 shows the results of all necessary pairwise comparisons.

**Table 2.** Summary of Pairwise Comparisons for Perspective Angle.

Comparison pair	Fixed factor(s)			Mean difference	95% CI	*p*-value
Magnified versus naked eye(s)	Ground	Diverged	All NE	−2.600	[−3.916,−1.284]	**<.001**
		Parallel		−1.097	[−2.402,−0.209]	**.016**
		Converged		−2.066	[−3.223,−0.908]	**.001**
		More converged	Binocular	** − 2.600**	[−3.916,−1.284]	**<.001**
			Monocular	1.097	[−0.209,2.402]	.095
	Sidewall	All ST	All NE	−1.544	[−2.100,−0.989]	**<.001**
Ground versus sidewall	All ST	All NE	Magnified	−0.278	[−1.333,0.777]	1.000
Ground versus ceiling				−2.132	[−3.853,−0.411]	**.012**
Sidewall versus ceiling				−1.854	[−3.409,−0.300]	**.016**

Significant differences (*p* <.05 ) are Bonferroni corrected. The comparisons are shown only in cases where post-hoc analysis can be performed.

ST = stimulus type; NE = number of eyes used.

### Optical Slant Angle

For optical slant angle, we also conducted a four-way ANOVA, but no significant four-way interaction was found. We then examined the simple interactions between pairs of factors. The results of simple interaction indicated significant interactions in LO × VC, LO × ST, and VC × ST. Similar to Table 1, only results up to the level with significant effects are included in Table 3 to avoid unnecessary clutter.

**Table 3.** Summary of Four-Way Repeated Measure ANOVA for Optical Slant Angle.

Analysis level	Effect	Sum of squares	*df*	Mean square	*F*-value	*p*-value
*Four-way ANOVA*						
	LO × NE	43.444	2	21.722	0.212	.810
	LO × VC	13079.374	1.596	8196.545	13.167	**<.001**
	NE × VC	0.224	1	0.224	0.002	.965
	LO × ST	1719.496	3.085	557.402	4.859	**.004**
	NE × ST	421.376	3	140.459	2.889	.042
	VC × ST	1019.206	3	339.735	4.746	**.005**
	LO × NE × VC	134.616	2	67.308	0.732	.487
	LO × NE × ST	465.385	6	77.564	1.355	.238
	LO × VC × ST	857.699	3.070	279.416	2.503	.065
	NE × VC × ST	257.613	3	85.871	2.036	.117
	LO × NE × VC × ST	281.377	3.520	79.928	1.095	.362

Significant differences ( p<.05 ) are Bonferroni corrected. Greenhouse-Geisser correction was applied where necessary.

ANOVA = analysis of variance; LO = location; VC = viewing condition; NE = number of eyes used; ST = stimulus type.

As we are primarily interested in the effect of VC and LO, we proceeded with pairwise comparisons for the LO × VC interaction. The results of these pairwise comparisons are presented in Table 4.

**Table 4.** Summary of Pairwise Comparisons for Optical Slant Angle.

Comparison Pair	Fixed Factor(s)			Mean Difference	95% CI	*p*-Value
Magnified versus naked eye(s)	Ground	All ST	All NE	−6.636	[−10.036,−3.236]	**<.001**
	Sidewall			−7.470	[−12.244,−2.696]	**.004**
	Ceiling			−21.638	[−28.747,−14.529]	**<.001**
Ground versus sidewall	Magnified	All ST	All NE	0.136	[−7.029,7.301]	1.000
Ground versus ceiling				**17.820**	[8.004,27.635]	**<.001**
Sidewall versus ceiling				**17.683**	[7.972,27.395]	**<.001**
Ground versus sidewall	Naked eye(s)	All ST	All NE	−0.698	[−5.814,4.418]	1.000
Ground versus ceiling				2.817	[−1.454,7.089]	.305
Sidewall versus ceiling				3.515	[−3.463,10.494]	.616

Significant differences ( p<.05 ) are Bonferroni corrected. The comparisons are shown only in cases where post-hoc analysis can be performed.

ST = stimulus type; NE = number of eyes used.
